# Daily Application of a Toothpaste with Biomimetic Hydroxyapatite and Its Subjective Impact on Dentin Hypersensitivity, Tooth Smoothness, Tooth Whitening, Gum Bleeding, and Feeling of Freshness

**DOI:** 10.3390/biomimetics5020017

**Published:** 2020-04-28

**Authors:** Sonja Steinert, Kai Zwanzig, Helga Doenges, Joern Kuchenbecker, Frederic Meyer, Joachim Enax

**Affiliations:** 1 Dental practice, Bielefeld 33602, Germany; heckel.sonja@gmx.net (S.S.); info@praxis-zwanzig.de (K.Z.); 2 Dental practice, Frankfurt am Main 60594, Germany; helga@doenges-frankfurt.de; 3 Dental practice, Bad Harzburg 38667, Germany; joernkuchenbecker@hotmail.com; 4 Dr. Kurt Wolff GmbH & Co. KG, Research Department, Bielefeld 33611, Germany; frederic.meyer@drwolffgroup.com

**Keywords:** teeth, biomimetic hydroxyapatite, oral care, toothpaste, dentin hypersensitivity, observational study

## Abstract

The aim of this observational study was to analyze the effect of a toothpaste with biomimetic zinc hydroxyapatite (HAP) on subjective parameters after a four-week home use. Patients with subjective dentin hypersensitivity were recruited at three dental practices in Germany and received a questionnaire with visual analogue scales and Likert scales both at baseline and follow-up. The questionnaire was specifically developed for this study and focused on questions about subjective parameters like dentin hypersensitivity, tooth surface texture, tooth color, and freshness after toothbrushing. Patients answered the questionnaire both at baseline and after a four-week home use (follow-up) of the HAP toothpaste. Data of 46 patients were analyzed by paired *t*-test and non-parametric Wilcoxon signed-rank test, respectively. Subjective parameters on dentin hypersensitivity were reduced after the four-week use of the HAP toothpaste (*p* < 0.001). Additionally, patients assessed their tooth surface as smoother (*p* < 0.001), tooth color as whiter (*p* = 0.003), and reported a stronger feeling of freshness after toothbrushing (*p* = 0.014) after four-week use of the HAP toothpaste compared to the previously used toothpaste. In conclusion, the tested toothpaste with biomimetic HAP is well-suited for individuals suffering from dentin hypersensitivity, because subjective symptoms on dentin hypersensitivity were reduced. Additionally, patients reported smoother and whiter teeth after using the HAP toothpaste.

## 1. Introduction

Toothpastes have been developed and continually improved for several decades to promote oral health [1]. It is well-known that both dental caries and gingivitis are caused by microbial biofilms present on the tooth surface [2]. Out of different preventive measures, toothbrushing is a key element to prevent those oral diseases [1]. Especially the toothpaste plays an important role, because it supports the mechanical plaque removal of the toothbrush by abrasives such as hydrated silica and calcium carbonate [1,3,4]. Additionally, the toothpaste provides various active ingredients for remineralization and plaque control. This includes, for example, fluorides, calcium phosphates, antibacterial agents, surfactants, and others [1,4]. However, the concept of controlling microorganisms instead of killing to keep up the homeostasis is becoming more and more established [5,6]. An interesting approach in modern preventive oral health care is the use of biomimetic and bio-inspired strategies because of controlling microorganisms rather than killing them and, thus, keeping the homeostasis of the oral microorganisms [7,8,9]. The number of publications using biomimetic concepts, e.g., hydroxyapatite, in preventive oral care has increased in the last decade [7,10,11], starting with publications in the late 1980s, e.g., by Huettemann and Doenges [12] and Kani et al. [13]. These biomimetic concepts aim to mimic, e.g., the structure of human enamel crystallites and/or the chemical composition of enamel [9,14]. Thus, hydroxyapatite (Ca_5_(PO_4_)_3_(OH); HAP) is a prominent example as a biomimetic ingredient [11]. Clinical studies and in-situ studies have shown HAP’s efficacy in preventing dentin hypersensitivity [7,12,15,16,17] and caries [7,13,18,19,20], in addition to the improvement of gingival health [7,21,22,23], respectively. The advantages of HAP are the high biocompatibility (no risk of fluorosis) and, thus, its broad application as an active ingredient for all age groups, including infants and toddlers [7,18,24,25,26]. Especially in the field of prevention of dentin hypersensitivity, HAP has been shown to be effective [7,12,15,16,17]. Nevertheless, in general, most of the studies testing active toothpaste ingredients have been performed with a mostly selective study population. Consequently, it is important to demonstrate preventive effects of toothpastes within the general population. Additionally, subjective feelings from patients affected by dentin hypersensitivity will help to improve the compliance for the home use of toothpastes. However, the majority of published studies miss analyzing the subjective feelings of the patients (e.g., tooth smoothness and freshness after toothbrushing). Therefore, the aim of this observational study using a pre-post-design was to analyze possible changes of various subjective parameters after a four-week home use of a toothpaste with biomimetic HAP in patients suffering from dentin hypersensitivity using a specific questionnaire developed by the authors.

Our study hypotheses were:

(I) The HAP toothpaste reduces subjective parameters of dentin hypersensitivity more efficiently

→ than the toothpaste previously used by the patients (primary hypothesis).

(II) The HAP toothpaste has also more positive effects on the parameters of dental care

→ convenience, i.e., tooth surface structure, tooth color, gum bleeding, and feeling of freshness after
→ toothbrushing than the toothpaste previously used by the patients (secondary hypothesis).

## 2. Materials and Methods

### 2.1. Recruitment, Inclusion Criteria, and Demographic Data

This observational study was performed as an open-label, uncontrolled, pre-post-design study. Patients were recruited at three dental practices in Germany: Bielefeld (K. Zwanzig), Frankfurt am Main (H. Doenges), and Bad Harzburg (J. Kuchenbecker). The following inclusion and exclusion criteria were verified at all three dental practices:

Inclusion criteria:

- Age of ≥ 18 years
- Subjective dentin hypersensitivity (at least 1 tooth) after using an airflow
- Overall good oral hygiene status
- Written informed consent

Exclusion criteria:

- Severe periodontitis
- Severe erosion damage
- Untreated caries lesion(s)

(Note that no additional inclusion or exclusion criteria were used.)

Subjects were enrolled after tooth cleaning and clinical examination. In total, 48 patients were included into the study (dental practice K. Zwanzig: 27 patients, dental practice H. Doenges: 13 patients, and dental practice J. Kuchenbecker: 8 patients). Note that questionnaires of two patients were excluded from the statistical analysis, because it was not possible to assign these questionnaires to a specific subject number (i.e., here, the identical subject number was given to two subjects). One patient terminated the study prematurely after 10 days (i.e., before the study end). However, this patient answered both baseline and follow-up questionnaires and was consequently included into the statistical analysis. Two patients reported on the taste (too spicy) but completed the study. In total, 46 patients were included into the statistical analysis (Table 1).

### 2.2. Toothbrushing at Home

Patients were asked to brush their teeth at home twice a day (in the morning and in the evening after meals) for 28 days with a commercially available HAP toothpaste (Wolff’s Biorepair Zahncreme, Dr. Kurt Wolff GmbH & Co. KG, Bielefeld, Germany). Toothpastes were provided by Dr. Kurt Wolff GmbH & Co. KG in commercially available packaging. Biorepair toothpaste is fluoride-free and contains 20% microcrystalline zinc HAP as the active ingredient [14,27], as well as the following other ingredients (according to the International Nomenclature of Cosmetic Ingredients, INCI):

Aqua, Zinc Hydroxyapatite, Hydrated Silica, Glycerin, Sorbitol, Silica, Aroma, Cellulose Gum, Sodium Myristoyl Sarcosinate, Sodium Methyl Cocoyl Taurate, Tetrapotassium Pyrophosphate, Zinc PCA, Sodium Saccharin, Phenoxyethanol, Benzyl Alcohol, Propylparaben, Methylparaben, Citric Acid, Sodium Benzoate.

The average brushing time was recommended to be ≥ 2 min. Patients were asked to continue using their favorite toothbrush, and no further oral care instructions were given (i.e., an influence of a new toothbrush and/or a new brushing technique, etc. can be excluded).

### 2.3. Questionnaires

Printed questionnaires (developed by the authors) were filled out at home by the patients themselves at baseline (i.e., before starting to use the HAP toothpaste) and after a 4-week home use of the HAP toothpaste (follow-up).

Due to the highly subjective parameters, patients answered the questions by using visual analogue scales (VAS) ranging from 0 to 100 mm (questions 1–6, 8, and 10) and Likert scales (questions 7 and 9). For the statistical analysis, the values were measured using a ruler (in millimeters). Note that the used VAS did not contain any measurement units visible for the patient. Questions concerning dentin hypersensitivity (questions 1–5) were based on frequently reported complaints which often occurred in this patient group [28].

The following questions were asked at baseline and again after a 4-week use of the HAP toothpaste (translated into English from German):

**Q1.**  How do you assess your tooth sensitivity in cold environments (cold beverages/meals, cold air, etc.)?

(VAS: No pain (0 mm), Very severe pain (100 mm))

**Q2.**  How do you assess your tooth sensitivity in sweet/acidic environments?

(VAS: No pain (0 mm), Very severe pain (100 mm))

**Q3.**  How do you assess your tooth sensitivity during toothbrushing?

(VAS: No pain (0 mm), Very severe pain (100 mm))

**Q4.**  Does your tooth sensitivity affect your daily life?

(VAS: Not at all (0 mm), Very strongly (100 mm))

**Q5.**  Do you have the feeling that your currently used toothpaste has reduced your tooth sensitivity?

(VAS: Much lower pain sensitivity (0 mm), Much higher pain sensitivity (100 mm))

**Q6.**  How do you assess the surface of your teeth after brushing with your toothpaste (by using the tongue)?

(VAS: Smooth (0 mm), Rough (100 mm))

**Q7.**  How long does the modified tooth surface feeling continue after toothbrushing?

(Options: 0 h/0.5 h/1 h/3 h/6 h/12 h)

**Q8.**  How do you assess the color of your teeth?

(VAS: White (0 mm), Yellowish/Brownish (100 mm))

**Q9.**  How often do you suffer from gum bleeding (average)?

(Options: Never/Less than 1 × month/1–3 × month/1–3 × week/4–6 × week/daily)

**Q10.**  How do you assess the feeling of freshness after toothbrushing?

(VAS: Very fresh (0 mm), Not fresh at all (100 mm))

Additionally, questions on the daily oral hygiene habits were asked, e.g., currently used toothpaste, toothbrush, and mouth rinse. The answers of all patients were transferred into a spreadsheet (Microsoft Excel, Redmond, WA, USA) after the study. This spreadsheet was used for the further statistical analysis.

### 2.4. Statistical Analysis

The statistical analysis was performed by Theodor W. May (Society for Biometry and Psychometry, Bielefeld, Germany). Paired *t*-test (questions Q1–Q6, Q8, and Q10) and non-parametric Wilcoxon signed-rank test (questions Q7 and Q 9) were used for statistical analyses. In addition, the Wilcoxon signed-rank test was performed for questions Q1–Q6, Q8, and Q10. Due to the explorative nature of this study, no adjustment of α-error for multiple testing was performed. Thus, all *p*-values should be interpreted as exploratory *p*-values (and not as confirmative *p*-values). In single cases, the total number of answers did not add up to *n* = 46 due to missing values. Missing values were not replaced.

## 3. Results and Discussion

### 3.1. Dental Care of Patients at Baseline

The patients mainly used toothpastes from international companies before participating in this study (Table 2).

### 3.2. Overview

This study indicates that subjective dentin hypersensitivity in cold, sweet, and/or acidic conditions decreased significantly after using the HAP toothpaste compared to the previously used toothpaste (*p* < 0.001; Table 3). Furthermore, the patients assessed the surfaces of their teeth as smoother, the color of the teeth as whiter, and reported a better freshness after toothbrushing at the follow-up questionnaire (i.e., after using the HAP toothpaste). There was no significant change of the frequency of bleeding gums. However, it should be noted that the cohort was not generally affected by bleeding gums (approx. 70% of the patients never had bleeding gums or less than once a month).

#### 3.2.1. Questions Relating to Dentin Hypersensitivity

The four-week use of the HAP toothpaste significantly reduced the subjective feeling of dentin hypersensitivity (Table 3 and Table 4). At baseline, the main causes of dentin hypersensitivity were cold stimulus, followed by sweet and/or acid stimulus. This result goes in line with another study using questionnaires, where cold stimulus was also reported to be the most significant factor for dentin hypersensitivity (89.3% of the patients, followed by toothbrushing (38.6%), hot (37.9%), and sweet (25%) stimuli) [28]. The HAP toothpaste reduced various subjective parameters (e.g., cold stimulus) on dentin hypersensitivity significantly better compared to previously used toothpastes (Table 3). Reducing the dentin hypersensitivity had also a positive influence on the quality of life (Table 4, question 4). These results from this cohort go in line with previously published clinical studies and show the efficacy of HAP in reducing dentin hypersensitivity with different patient groups [7,12,15,16,17,29,30,31]. A recent systematic review and meta-analysis shows that HAP as the active ingredient relieves symptoms of dentin hypersensitivity more efficiently than negative controls [15]. Orsini et al., for example, showed in a clinical trial that a hydroxyapatite-containing toothpaste effectively reduced dentin hypersensitivity after four and eight weeks. The efficacy was comparable to a desensitizing toothpaste with sodium fluoride/potassium nitrate [17]. Mechanistic studies on HAP toothpastes in the field of dentin hypersensitivity were reported, e.g., by Hiller et al. [32] and Amaechi et al. [33]. The mode of action of HAP in the prevention of dentin hypersensitivity is based on the occlusion of open dentin tubules by HAP particles [7]. Microcrystalline HAP is able to occlude dentin tubules that are known to have a diameter (superficial) of about 2.4 ± 0.07 µm [34]. Occlusion of dentin tubules leads to an inhibition of the cascade from external stimuli directly to the pulp, thus decreasing the feeling of pain [31].

#### 3.2.2. Questions Relating to Tooth Surface Texture, Tooth Color, and Feeling of Freshness

Patients reported positive changes on tooth surface texture, tooth color, and the feeling of freshness after four-weeks’ use of HAP toothpaste compared to baseline (Table 5). The use of the HAP toothpaste leads to the long-lasting feeling of a smooth tooth surface (assessed by the patient’s tongue) (Table 6). This finding indicates a possible mode of action of HAP on the diminished adhesion of bacteria to tooth surfaces [35,36]. Thus, the smoother tooth surface that was reported by the patients may reduce the bacterial adhesion and, also, the adhesion of stains [37]. The subjective feeling of tooth-smoothing can be explained by the reduction of bacterial colonization by HAP particles [36,38] and the remineralization/repair of small enamel defects [14,19,20,39,40,41,42], as well as the cleaning efficacy of the toothpaste itself [1,3]. HAP may act as a “filler” for micropores in the enamel [1]. Micropores can occur because of abrasive food, highly abrasive whitening toothpastes (e.g., toothpastes for smokers), bruxism, or acidic attacks. By filling those pores, biomimetic HAP may smoothen the tooth surface. To the best of the authors’ knowledge, this is the first study under in-vivo conditions that analyzed and confirmed the subjective feeling of tooth smoothness after using a HAP toothpaste. Additionally, patients reported a subjective feeling of whiter teeth and freshness. The whitening properties of HAP-based oral care formulations have been previously published; see, e.g., references [37,43,44,45,46]. In contrast, stannous salts, which are frequently used in oral care formulations/products against dentin hypersensitivity (see, e.g., reference [47]), may stain the tooth surface [48,49] and, understandably, may diminish patients’ compliance.

#### 3.2.3. Questions Relating to Gum Bleeding

While another 12-week clinical study with patients with mild-to-moderate periodontitis showed a reduction in gum bleeding [22], there was no change with respect to gum bleeding in this cohort (Table 7). This could be explained by the fact that most of the patients (approx. 70%) did not show any gum bleeding at all or were less than once a month affected by gum bleeding (Table 7).

## 4. Conclusions

This study showed that subjective parameters of dentin hypersensitivity (e.g., induced by cold, sweet, and/or acid stimuli) in a general population were reduced (*p* < 0.001) after the four-week use of a toothpaste with biomimetic HAP. Additionally, patients assessed their tooth surface as smoother (*p* < 0.001), tooth color as whiter (*p* = 0.003), and reported a stronger feeling of freshness (*p* = 0.014) after the four-week use of the HAP toothpaste compared to baseline. The toothpaste with biomimetic HAP is well-suited for the everyday use for individuals suffering from dentin hypersensitivity. The HAP toothpaste reduced subjective symptoms of dentin hypersensitivity and led to a subjective feeling of smoother and whiter teeth. Compared to other oral care ingredients, the key advantages of biomimetic HAP are its multifunctionality, as well as its similarly to human enamel crystallites and, consequently, its high biocompatibility.

## Figures and Tables

**Table 1:** Gender and age of the study population.

**Gender**	**Female**		**Male**		**Total**			
	n	%	n	%	n		%	
	34	73.9%	12	26.1%	46		100.0%	
**Age Distribution of the Patients**	Mean	SD	P25%	Median	P75%	Min	Max	N
	44.9	13.0	34.0	43.0	55.0	23	68	45

SD, standard deviation; P25%, 25% percentile; and P75%, 75% percentile.

**Table 2:** Overview of toothpastes, toothbrushes, and other dental care products used by the patients before baseline.

		*n*	%
**Toothpaste Used** **(Before Using the HAP Toothpaste)**	Elmex, Aronal, Meridol (CP GABA GmbH, Hamburg, Germany)	15	36.6%
	Colgate (CP GABA GmbH, Hamburg, Germany)	4	9.8%
	Sensodyne (GlaxoSmithKline Consumer Healthcare GmbH & Co. KG, Munich, Germany)	4	9.8%
	Signal (Unilever Deutschland GmbH, Hamburg, Germany)	3	7.3%
	Odol-med 3 (GlaxoSmithKline Consumer Healthcare GmbH & Co. KG, Munich, Germany)	6	14.6%
	Other	9	22.0%
	Total	41	100.0%
**Electric or Manual Toothbrush**	Electric toothbrush	22	47.8%
	Manual toothbrush	17	37.0%
	Both	7	15.2%
	Total	46	100.0%
**Toothbrush**	Phillips (Philips GmbH, Hamburg, Germany)	4	12.1%
	Colgate (CP GABA GmbH, Hamburg, Germany)	1	3.0%
	Dr. Best (GlaxoSmithKline Consumer Healthcare GmbH & Co. KG, Munich, Germany)	2	6.1%
	Oral B (Procter & Gamble, Schwalbach, Germany)	14	40.0%
	Other	12	34.3%
	Various	2	5.7%
	Total	35	100.0%
**Additional Oral Care Products**	1 product	15	34.9%
	2 products	16	37.2%
	3 products	10	23.3%
	4 products	2	4.7%
	Total	43	100.0%
**Mouth Rinse**	Elmex, Aronal, Meridol (CP GABA GmbH, Hamburg, Germany)	2	22.2%
	Listerine (Johnson & Johnson GmbH, Neuss, Germany)	2	22.2%
	Other	5	55.6%
	Total	9	100.0%

**Table 3:** Significance tests between baseline and follow-up (after 4-week use of the HAP toothpaste); *p*-values of the *t*-test for paired samples for pre-post-changes. The non-parametric Wilcoxon tests confirm the results of the *t*-tests for paired samples.

Questions	Significant Effect After Using HAP Toothpaste
Q1. Tooth sensitivity in cold environments	Yes (*p* < 0.001)
Q2. Tooth sensitivity in sweet/acidic environments	Yes (*p* < 0.001)
Q3. Tooth sensitivity during toothbrushing	Not significant (*p* = 0.168)
Q4. Daily life affected by tooth sensitivity	Yes (*p* = 0.026)
Q5. Reduction of tooth sensitivity	Yes (*p* = 0.003)
Q6. Surface of teeth	Yes (*p* < 0.001)
Q7. Duration of smoothness	Yes (*p* < 0.001)
Q8. Color of teeth	Yes (*p* = 0.003)
Q9. Gum bleeding	Not significant (*p* = 0.170)
Q10. Freshness after toothbrushing	Yes (*p* = 0.014)

**Table 4:** Subjective parameters of dentin hypersensitivity at baseline and at follow-up (after 4-week use of HAP toothpaste).

**Baseline**	**Mean**	**SD**	**P25%**	**Median**	**P75%**	**Min**	**Max**	***n***
1. Tooth sensitivity in cold environments (0 = no pain, 100 = very severe pain)	60.3	23.1	50.0	64.5	78.0	12	100	46
2. Tooth sensitivity in sweet/acidic environments (0 = no pain, 100 = very severe pain)	35.3	28.5	9.0	32.5	55.0	0	100	46
3. Tooth sensitivity during toothbrushing (0 = no pain, 100 = very severe pain)	25.0	22.3	6.0	19.5	48.0	0	70	46
4. Daily life affected by tooth sensitivity (0 = not at all, 100 = very strong)	33.9	25.6	6.0	35.0	55.0	0	85	46
5. Reduction of tooth sensitivity by currently used toothpaste (0 = much less pain, 100 = much more pain)	43.1	17.3	37.0	49.0	51.0	0	75	44
**Follow-up**	**Mean**	**SD**	**P25%**	**Median**	**P75%**	**Min**	**Max**	***n***
1. Tooth sensitivity in cold environments (0 = no pain, 100 = very severe pain)	40.0	24.8	20.0	37.5	64.0	0	97	46
2. Tooth sensitivity in sweet/acidic environments (0 = no pain, 100 = very severe pain)	21.9	22.6	5.0	13.5	32.0	0	83	46
3. Tooth sensitivity during toothbrushing (0 = no pain, 100 = very severe pain)	20.9	21.8	4.0	14.0	34.0	0	97	45
4. Daily life affected by tooth sensitivity (0 = not at all, 100 = very strong)	27.5	24.0	5.0	24.0	50.0	0	72	46
5. Reduction of tooth sensitivity by currently used toothpaste (0 = much less pain, 100 = much more pain)	33.0	20.1	17.5	31.0	48.0	0	88	44

SD, standard deviation; P25%, 25% percentile; and P75%, 75% percentile.

**Table 5:** Subjective parameters of dental care convenience (tooth surface, color of teeth, and freshness after tooth-brushing) at baseline and at follow-up (after 4-week use of HAP toothpaste).

	**Mean**	**SD**	**P25%**	**Median**	**P75%**	**Min**	**Max**	***n***
6. Surface of teeth after brushing (0 = smooth, 100 = rough)	23.8	18.3	7.0	20.5	39.0	0	60	46
8. Color of teeth (0 = white, 100 = yellowish/brownish)	48.5	20.6	35.0	46.0	63.0	7	100	46
10. Freshness after toothbrushing (0 = very fresh, 100 = not fresh at all)	28.4	20.6	9.0	27.5	46.0	0	74	46
**Follow-up**	**Mean**	**SD**	**P25%**	**Median**	**P75%**	**Min**	**Max**	***n***
6. Surface of teeth after brushing (0 = smooth, 100 = rough)	14.2	15.0	4.0	10.0	21.0	0	71	46
8. Color of teeth (0 = white, 100 = yellowish/brownish)	43.9	19.2	29.0	43.0	55.0	4	88	45
10. Freshness after toothbrushing (0 = very fresh, 100 = not fresh at all)	20.6	15.6	4.0	20.0	29.0	0	63	45

SD, standard deviation; P25%, 25% percentile; and P75%, 75% percentile.

**Table 6:** Duration of modified tooth surface feeling (tooth smoothness after toothbrushing) at baseline and at follow-up (after 4-week use of HAP toothpaste).

	7. Duration of Modified Tooth Surface Feeling After Toothbrushing Baseline		7. Duration of Modified Tooth Surface Feeling After Toothbrushing Follow-up	
	*n*	%	*n*	%
0 h	4	8.9%	2	4.7%
0.5 h	5	11.1%	2	4.7%
1 h	13	28.9%	10	23.3%
3 h	16	35.6%	12	27.9%
6 h	6	13.3%	11	25.6%
12 h	1	2.2%	6	14.0%
Total	45	100.0%	43	100.0%

**Table 7:** Frequency of gum bleeding at baseline and at follow-up (after 4-week use of HAP toothpaste).

	9. Frequency of Gum Bleeding Baseline		9. Frequency of Gum Bleeding Follow-up	
	*n*	%	*n*	%
never	15	32.6%	16	35.6%
Less than 1 × month	17	37.0%	21	46.7%
1–3 × month	10	21.7%	4	8.9%
1–3 × week	3	6.5%	3	6.7%
4–6 × week	1	2.2%	1	2.2%
daily	0	0.0%	0	0.0%
Total	46	100.0%	45	100.0%

The sample size is smaller than *n* = 46 because of missing or not evaluable information.
